# Neural networks determination of material elastic constants and structures in nematic complex fluids

**DOI:** 10.1038/s41598-023-33134-x

**Published:** 2023-04-13

**Authors:** Jaka Zaplotnik, Jaka Pišljar, Miha Škarabot, Miha Ravnik

**Affiliations:** 1 Faculty of Mathematics and Physics, University of Ljubljana, 1000 Ljubljana, Slovenia; 2 Jožef Stefan Institute, 1000 Ljubljana, Slovenia

**Keywords:** Characterization and analytical techniques, Condensed-matter physics, Structure of solids and liquids, Liquid crystals

## Abstract

Supervised machine learning and artificial neural network approaches can allow for the determination of selected material parameters or structures from a measurable signal without knowing the exact mathematical relationship between them. Here, we demonstrate that material nematic elastic constants and the initial structural material configuration can be found using sequential neural networks applied to the transmmited time-dependent light intensity through the nematic liquid crystal (NLC) sample under crossed polarizers. Specifically, we simulate multiple times the relaxation of the NLC from a random (qeunched) initial state to the equilibirum for random values of elastic constants and, simultaneously, the transmittance of the sample for monochromatic polarized light. The obtained time-dependent light transmittances and the corresponding elastic constants form a training data set on which the neural network is trained, which allows for the determination of the elastic constants, as well as the initial state of the director. Finally, we demonstrate that the neural network trained on numerically generated examples can also be used to determine elastic constants from experimentally measured data, finding good agreement between experiments and neural network predictions.

## Introduction

Machine learning (ML) methods are increasingly used in different contexts of materials physics, such as for the discovery of new materials with desired properties^1,2^, for the identification of phases, phase transitions^3,4^ and order parameters for several Hamiltonians^5^. In suspensions of active Brownian particles, the belonging of single particles to possible phases can be predicted from their individual features using artificial neural networks^6^. ML is utilized in modeling of structures of self-assembled lipids^7^, characterizing 3-dimensional colloidal systems^8^, analysis of complex local structure of liquid crystal polymers^9^, acceleration of simulations of fluids^10^ and other soft matter, for example active matter^11^ including active nematics^12–14^. Deep learning algorithms are also becoming useful analytical tools for microscopic image analysis^15,16^ and micro-praticle tracking^17^. Neural networks can be employed to estimate Reynolds number for flows around cylinders^18^ and also for drag prediction of arbitrary 2D shapes in laminar flow at low Reynolds number^19^. ML algorithms can be exploited to determine the order parameter, the temperature of a sample^20^, phases^21^ and phase transition temperatures^22^ of liquid crystals, and also pitch lengths of cholesteric liquid crystals^23^ from polarized light microscopy images as well as to identify types of topological defects in NLCs from the known director field^24^ or to predict the specific heat of newly designed proteins^25^. ML algorithms, specifically linear support vector machines, have also been employed as classifiers to optimize automated liquid crystal-based chemical sensors^26^. Furthermore, by combining observation of liquid crystal droplets and machine learning, it is possible to identify and quantify endotoxins from different bacterial species^27^.

Soft matter equilibrium is at the mesoscopic level determined by the minimum of the total free energy, and for nematic complex fluids, the leading—elastic—free energy is determined by three elastic constants $K_{11}$, $K_{22}$, and $K_{33}$ that are attributed to the three fundamental elastic modes: splay, twist, and bend, correspondingly. The common and established techniques for measuring the elastic constants are based on Fréedericksz transition^28^, where the abrupt change of the molecular ordering director field can be detected by optical or calorimetric measurements. Typically, a specific cell is needed to measure a distinct elastic constant; however, methods that include hybrid cells allow measurements of all three elastic constants simultaneously^29^. The measurement can also be done all-optically using polarized laser beams that induce optical Fréedericksz transition^30^ or by comparing structural transitions in experimental samples and numerically simulated cholesteric LC droplets under electric field^31^. More challenging than the measurement of material parameters, such as elastic constants, can be the recognition of the liquid crystal director structure. Full three-dimensional spatial liquid crystal orientational profiles can be determined from angular dependence of fluorescence in nematics using fluorescent confocal polarising microscopy (FCPM)^32,33^ or alternatively, the dielectric tensor and the corresponding director field can be reconstructed by tomographic approaches^34^.

In this study, we develop a neural networks-based method for the determination of elastic constants and simple nematic structures from standard light transmittance measurement of a confined nematic sample between crossed polarizers during the dynamical relaxation of the nematic to the minimum free energy state from an arbitrary initial state, such as induced by random electric field. Specifically, the elastic constants are determined by an artificial neural network (ANN) that is trained beforehand on thousands of pairs of numerically simulated transmittances for various initial states and random elastic constants. The method is validated against full experiments measurement data, finding very good agreement between predicted and actual elastic constants. Complementary, also the method can yield the initial configuration of the director field from the time-dependent transmittance after or if the elastic constants are known.

**Figure 1:**
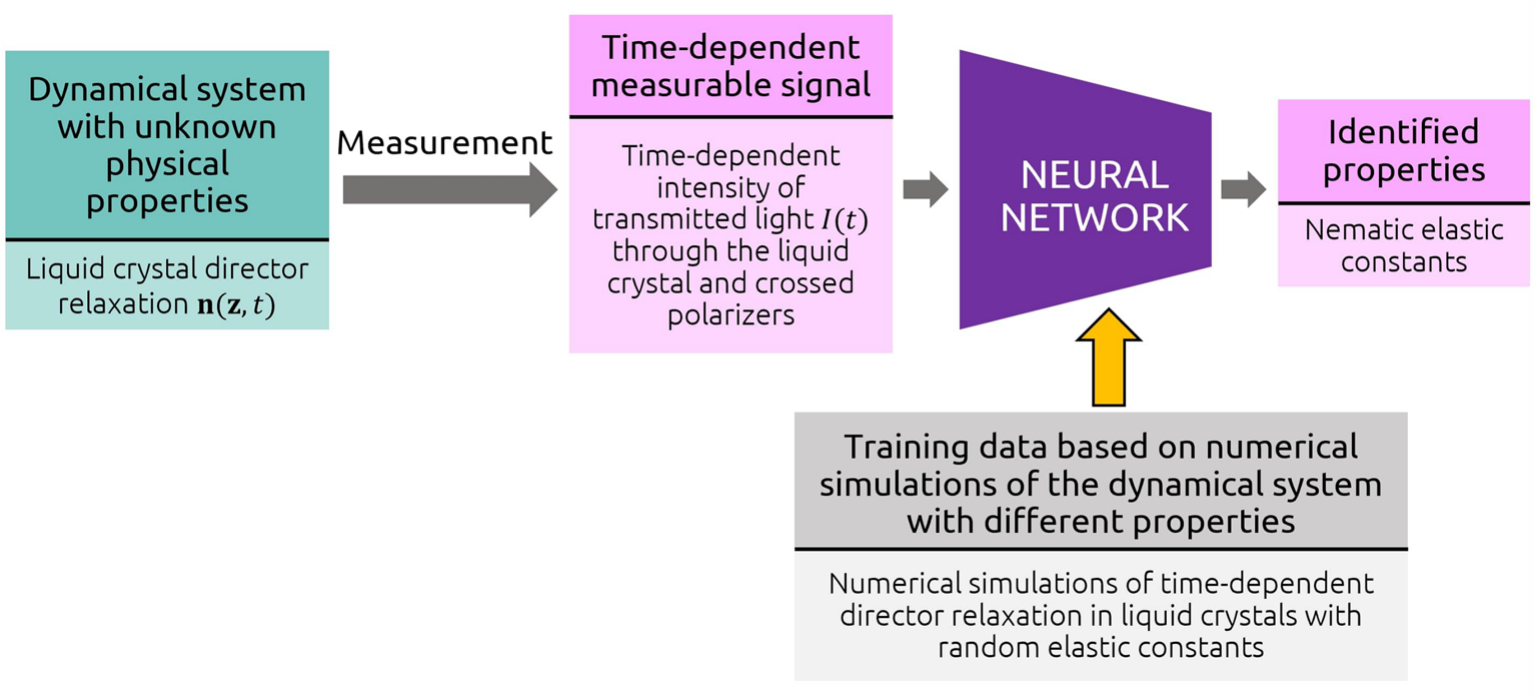
Graphical overview of the method for the identification of unknown parameters of dynamical systems based on numerical simulations and artificial neural networks. In this study, such an approach was used to determine Frank nematic elastic constants from measured time-dependent intensities of transmitted light through liquid crystals.

## Results

The developed neural networks-based method for determining elastic constants is based on the combined modeling of (i) liquid crystal effective dynamics, (ii) light transmission, and (iii) supervised machine learning, as then applicable both to experimental or modeling data. The method starts by calculating a large number of time-dependent light beam transmittance functions *I*(*t*) during the relaxation of the NLC sample that correspond to different elastic constants, which we then use to train the neural network that recognizes elastic constants from a simulated measurement. Later, the well-trained neural network is used to predict elastic constants also from signals measured from real samples in the laboratory. A general overview of the method, which could, in principle, be used for any other experimentally relevant setup and determining other material parameters, is shown in Fig. 1. Simulation of LC profiles is explained in Methods.

### Neural networks based method for determining elastic constants

The orientational dynamics of a nematic liquid crystal depends on the initial state, rotational viscosity $\gamma _1$, cell thickness *D* and also on Frank elastic constants. Therefore, if the director field is initially deformed by short pulses of the electric or magnetic field, it will reconfigure back to the equilibrium state after the fields are turned off. Consequently, the intensity of the transmitted light through the liquid crystal cell between the crossed polarizers also varies during the relaxation and its time-dependence therefore indirectly carries information about the elastic constants. So the idea of this method is to use a neural network to identify elastic constants from the time-dependent intensities of transmitted light, regardless of the initial state of the director. However, to perform this task, the neural network must be trained on data, i.e. on many examples of time-dependent intensities *I*(*t*) and associated elastic constants. In the setup for the determination of the nematic elastic constants, we assume a specific cell geometry where the liquid crystal is confined in a thin cell of thickness $D\sim 10\ \upmu \text {m}$ with strong anchoring which is uniform in *x* and *y* directions on both boundaries, so that the director, varies only along the *z* axis and in time *t*, $\textbf{n}= \textbf{n}(z,t)=(n_x(z,t),n_y(z,t),n_z(z,t))=(\cos \phi (z,t)\cos \theta (z,t),\sin \phi (z,t)\cos \theta (z,t),\sin \theta (z,t))$, where $\theta \in [-\pi /2,\pi /2]$ and $\phi \in [-\pi ,\pi ]$ are spherical angles. For determining only splay and bend constants, $K_{11}$ and $K_{33}$, even more simple 2D director geometry (i.e. director profile variability), $\textbf{n}(z,t)=(\cos \theta (z,t),0,\sin \theta (z,t))$, proves sufficient.

**Figure 2:**
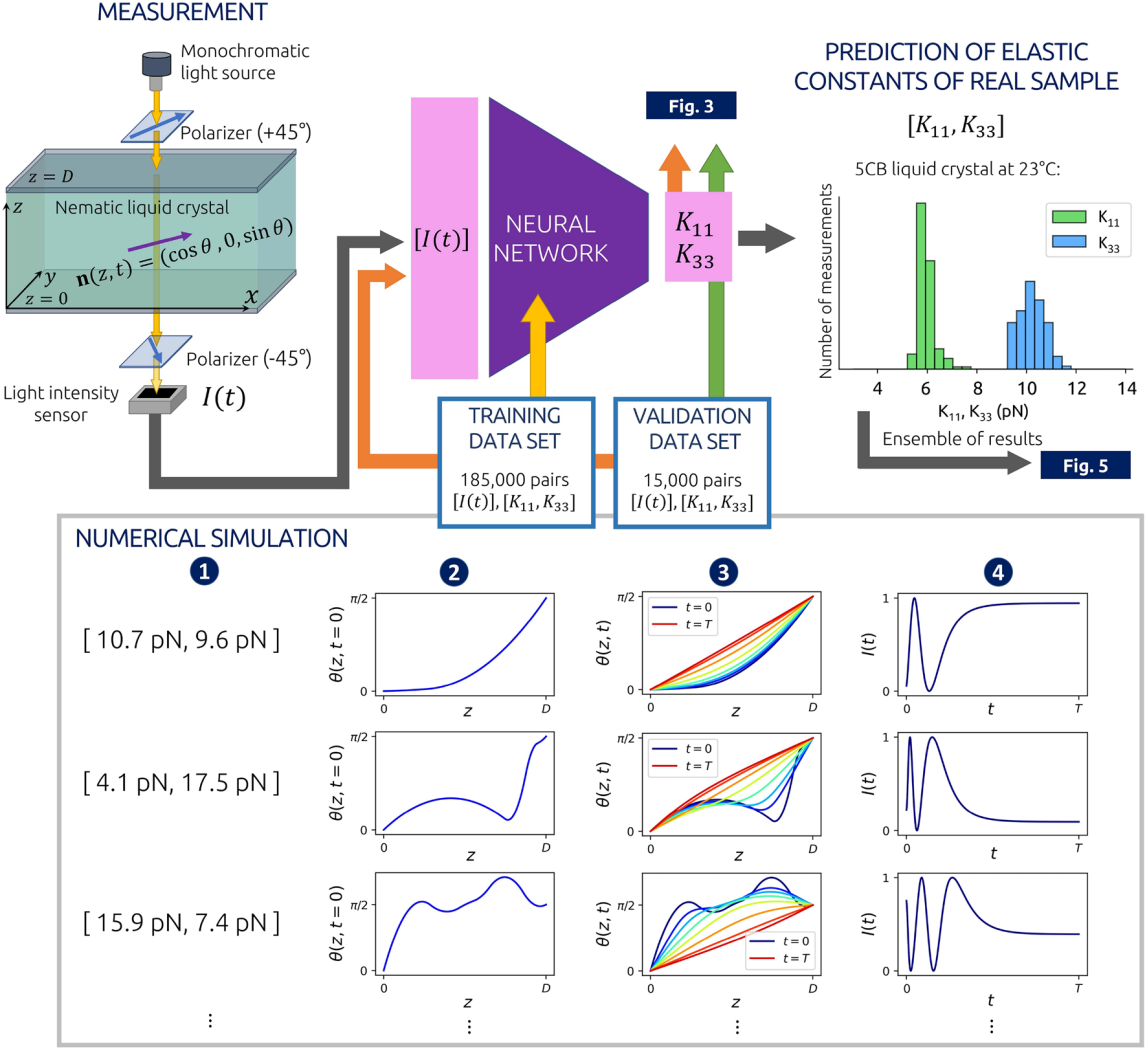
Scheme of neural network-based method for determining the elastic constants of splay ($K_{11}$) and bend ($K_{33}$) deformations. First, the elastic constants are randomly set (1), then the random initial state of the director, $\textbf{n}(z,t=0)$, is generated (2), and accordingly, the dynamics of the director $\textbf{n}(z,t)$ is calculated (3) that allows us to simulate the time dependence of the intensity of transmitted light through the sample between crossed polarizers (4). This is repeated 200,000 times to generate training and a validation data set. Once the neural network is well-trained, the experimentally measured intensity *I*(*t*) can be used to determine the elastic constants of a real liquid crystal sample. For example, for the determination of elastic constants of 5CB, the cell thickness was $D=10\ \upmu$m and the time interval was $T=1.28$ s.

#### Determining $K_{11}$ and $K_{33}$

The scheme of the method for determining $K_{11}$ and $K_{33}$ is shown in Fig. 2. To create a training set, we start with generating a pair of random elastic constants $K_{11}$ and $K_{33}$ from the uniform distribution in the interval of possible expected values. For example, to predict the elastic constants of 5CB liquid crystal at approx 23 °C, we choose elastic constants from the uniform distribution $U(2\text { pN}, \ 18\text { pN})$ as we expect the actual constants somewhere within this interval^29,35,36^. Next, the initial non-equilibrium state of the director $\textbf{n}(z,t=0)=(\cos \theta (z,t=0),0,\sin \theta (z,t=0))$ is set by generating a random, not necessarily physically meaningful smooth 1D function $\theta (z,t=0)$ for $z\in [0,D]$ by quadratic interpolation between a random number of points at different random positions within the interval [0, *D*]. Having the initial state $\textbf{n}(z,t=0)$, a pair of elastic constants and the rotational viscosity $\gamma _1$ (e.g. $0.098 \text { Pa s}$ for 5CB at the room temperature^37,38^), the dynamics $\textbf{n}(z,t)$ is numericallly simulated (see “Methods”). The boundary conditions $\textbf{n}(z=0,t)$ and $\textbf{n}(z=D,t)$ are determined by the selected anchoring type on each boundary. Different combinations of planar ($\theta =0$) and homeotropic ($\theta =\pi /2$) anchoring are tested. From the configuration of the director at each time step, the transmission of light through crossed polarizers and the sample of reconfiguring liquid crystal between them are calculated using the Jones matrix formalism^39,40^. We comment that more advanced light propagation methods could also be used, such as finite difference time domain (FDTD)^41^, but give no qualitative difference for the considered nematic geometries. The values of the ordinary and extraordinary refractive indices, the thickness of the cell and the spectrum of the light source are required to be known precisely for particular liquid crystal material and experimental setting. In this way, the time dependence of the transmittance *I*(*t*) is calculated and discretized at, for example, 500 time steps for each pair of corresponding random elastic constants and the initial state of the director. Repeating this 200,000 times, a set of 200,000 pairs of input vectors ${\textbf{X}}_i=[I_i(t=0),I_i(t=\Delta t), ...,I_i(t=T=499\Delta t)]$ and expected (true) output vectors ${\textbf{T}}_i=\left[ \widetilde{K_{11}^i},\widetilde{K_{33}^i}\right]$, packed as $\{(\textbf{X}_1,\textbf{T}_1), ..., (\textbf{X}_{200000},\textbf{T}_{200000}) \}$, is obtained, which is later split into a training and a validation data set of lengths 185,000 and 15,000, respectively. The time of the interval *T* depends on the geometry and rotational viscosity $\gamma _1$, and we set it to such a value that the intensity *I*(*t*) saturates and effectively becomes constant. For training, the data is – as usual for neural network training^42^ – linearly scaled onto the [0, 1] interval. While the relative intensities *I*(*t*) are already limited to this interval by themselves, we transform elastic constants by the *“MinMaxScaler”* as $\widetilde{K_{11}^i}=\left( K_{11}^i - \min _j\left( K_{11}^j\right) \right) /\left( \max _j\left( K_{11}^j\right) -\min _j\left( K_{11}^j\right) \right)$ and analogously for $\widetilde{K_{33}}$ . The training data set is used to train the weights and biases of a dense sequential neural network so that the difference between the predicted output ${\textbf{Y}}$ and the expected output ${\textbf{T}}$ iteratively becomes as small as possible. To quantify the difference, the mean absolute error was used as a loss function. The validation set is used to test the neural network’s performance for data that was not used for training. Training via Adam optimization algorithm^43^ was performed using Tensorflow Keras software^44,45^ with batch size 25 and learning rate $\eta =0.0003$. A neural network with an input layer of 500 neurons and four hidden layers of 500, 400, 250, and 100 neurons and rectified linear unit (ReLU) activation functions and an output layer of two neurons with sigmoid activation functions was used. It turns out that the network’s architecture can be substantially varied as long as the total number of parameters (connections between neurons) is large enough (for our study, larger than order-of-magnitude $\sim 10^{5}$). As observed, adding more layers or increasing the number of neurons in layers in the described model does not significantly improve its accuracy.

**Figure 3:**
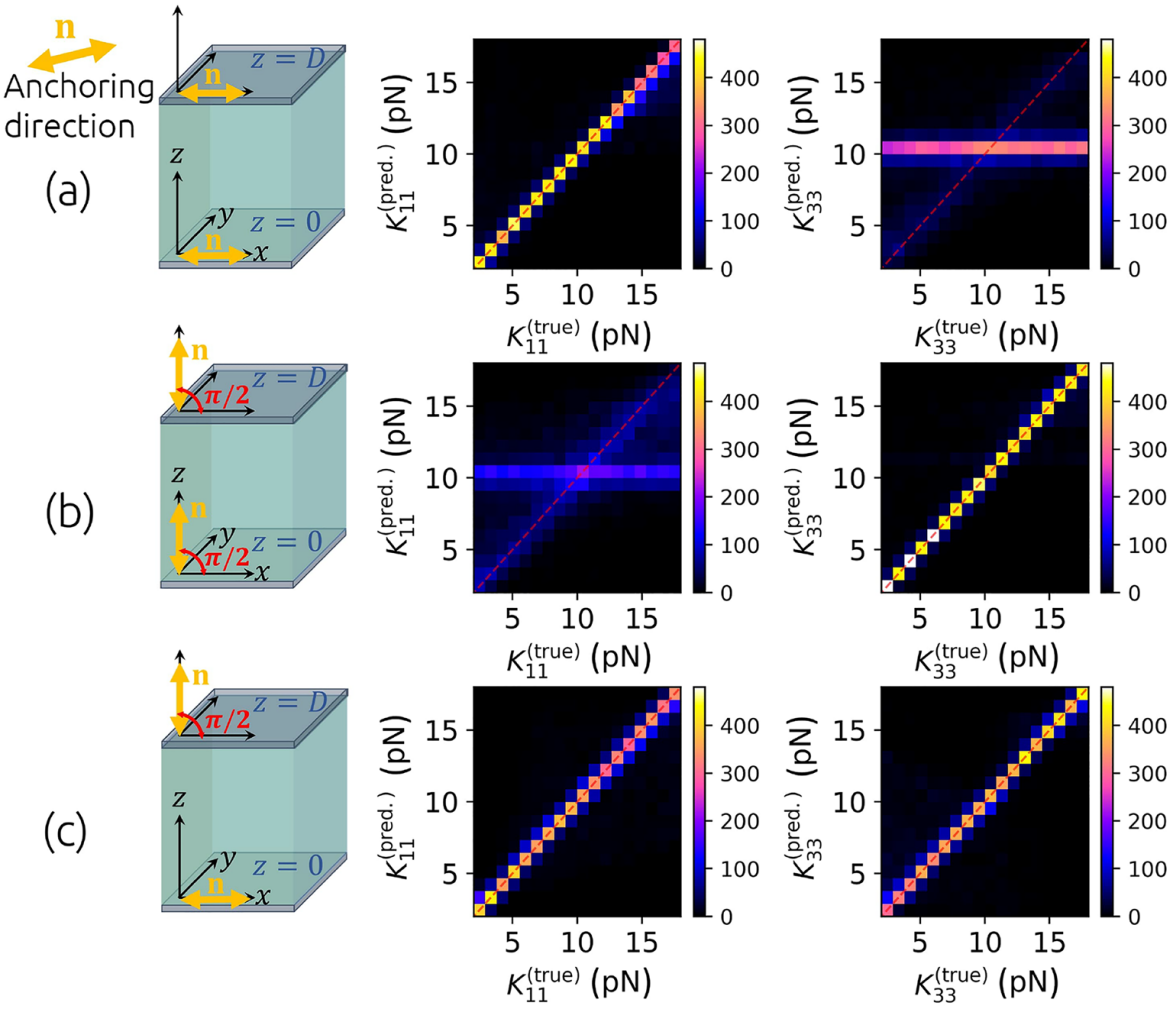
Comparison of predicted and actual elastic constants $K_{11}$ and $K_{33}$ from the validation data set with $\textbf{n}(z,t)=(\cos \theta (z,t),\ 0,\ \sin \theta (z,t))$ director geometry and three different combinations of anchoring. Planar-planar geometry (**a**) allows for precisely predicting $K_{11}$ but not $K_{33}$, using homeotropic-homeotropic geometry (**b**), $K_{33}$ can be determined, whereas in planar-homeotropic geometry (**c**), one can train a neural network to determine both elastic constants $K_{11}$ and $K_{33}$ at once. The planar-homeotropic configuration is later also used in experiments.

**Figure 4:**
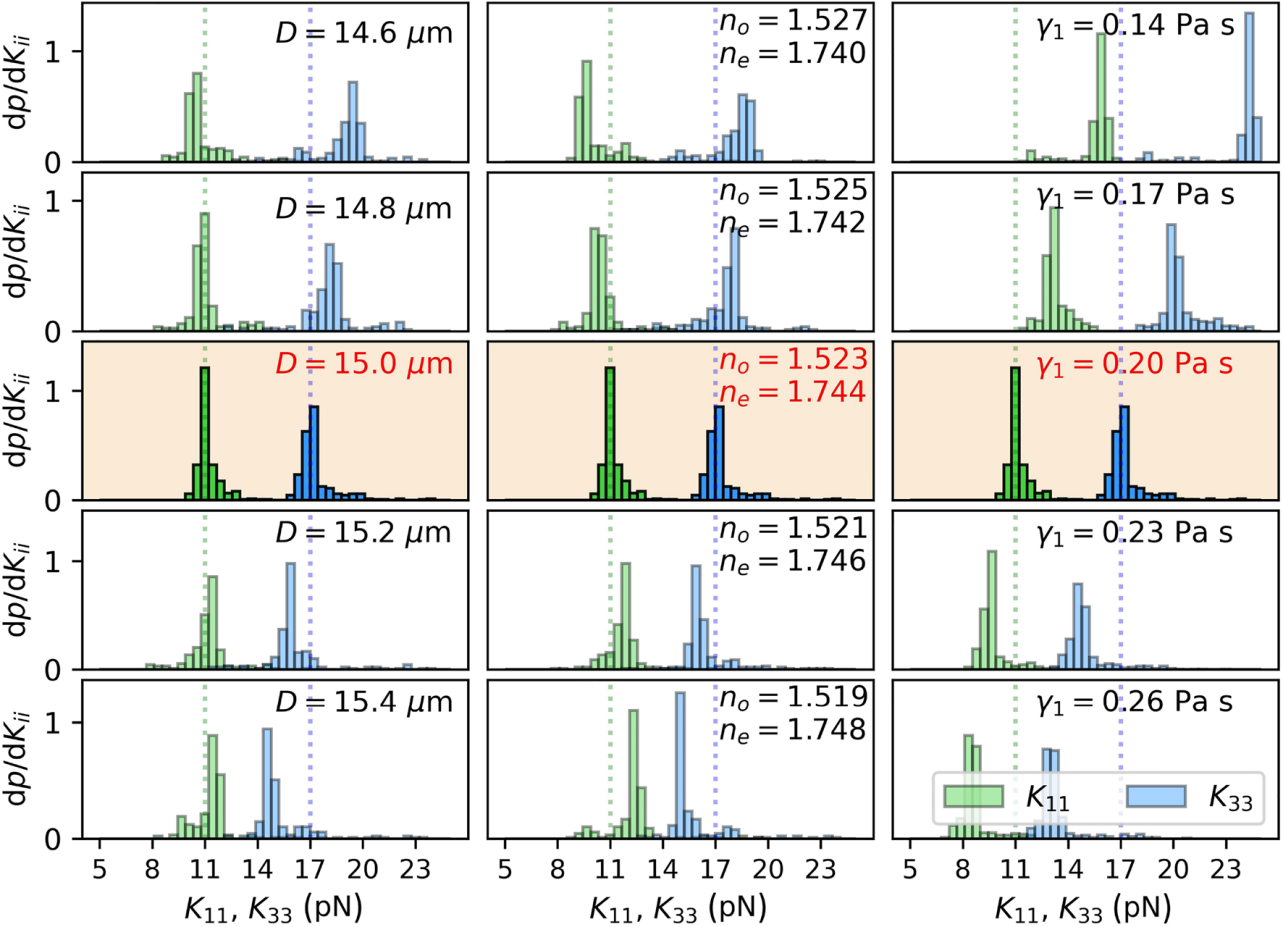
Sensitivity of the method for determining $K_{11}$ and $K_{33}$ to the inaccuracy of parameters used to generate the training data. The neural network that predicts $K_{11}$ and $K_{33}$ from *I*(*t*) is trained on data from simulations with $D=15\ \upmu \textrm{m}$, $\gamma _1=0.200 \ \text {Pa s},$ $n_o=1.523$, and $n_e=1.744$. If the neural network is used to predict elastic constants of a sample with actual constants $K_{11}=11 \text { pN}$, $K_{33}=17 \text { pN}$ and exactly the same parameters ($D,\ \gamma _1, \ n_o,\ n_e$), good quality of prediction is shown.

A neural network can be trained to recognize both elastic constants $K_{11}$ and $K_{33}$ from time-dependent transmittances *I*(*t*) through samples with $\textbf{n}(z,t)=(\cos \theta (z,t),0,\sin \theta (z,t))$ director geometry only if the planar ($\theta (z=0)=0$) and the homeotropic ($\theta (z=D)=\pi /2$) anchoring are used at the opposite cell surfaces. Planar-planar anchoring geometry allows us to determine $K_{11}$ only, while in cells with homeotropic anchoring on both surfaces, only $K_{33}$ can be determined. This is shown in Fig. 3, where 2D histograms show the number count of points in particular sections of the 2D space of true versus predicted constants for examples from the validation set. For a single type of anchoring on both plates, the equilibrium orientation of molecules is always constant along *z*-axis, either $\theta (z,t\rightarrow \infty )=0$ or $\theta (z,t\rightarrow \infty )=\pi /2$, regardless of the elastic constants. Consequently, the transmittance after relaxation, $I(t\rightarrow \infty )$, does not depend on them and therefore the information about elastic constants is efffectively embedded in the effective dynamics of the time-dependent *I*(*t*). In the planar-planar geometry, the dynamics in the last part of the relaxation, when the deformations are small, and the director is only slightly different from its equilibrium configuration $\textbf{n}(z,t\rightarrow \infty )=(1,0,0)$, is mainly governed by $K_{11}$. This follows from the fact that in a small-deformations regime, $(\partial n_z/\partial z)^2\gg (\partial n_x/\partial z)^2$ and therefore the Frank-Oseen elastic free energy density (“Methods”: Eq. 1) simplifies to $f_{FO}\approx K_{11/2}(\partial n_z/\partial z)^2$. This is the reason why only $K_{11}$ can be determined in such geometry. In homeotropic-homeotropic geometry, it is just the opposite. The dynamics is governed by $K_{33}$ and therefore only $K_{33}$ can be determined. However, the equilibrium configuration of the director in a cell with planar-homeotropic anchoring geometry is described by $\theta (z,t\rightarrow \infty )=\pi z/2D$ if $K_{11}=K_{33}$ and by a convex or a concave function $\theta (z,t\rightarrow \infty )$ when $K_{11}>K_{33}$ or $K_{11}<K_{33}$, respectively. This results also in the dependence of $I(t\rightarrow \infty )$ on both elastic constants and for this reason, *I*(*t*) carries more information in such geometry and it is possible to determine both elastic constants at the same time. Consequently, such geometry has been chosen to be studied in more detail. As depicted by the red curve in Fig. 6, the mean absolute error of the predicted elastic constants can decrease down to $\overline{\sigma _{ K_{ii}}}\approx 0.5 \text { pN}$ after 60 epochs of training and batch size 25 using the training set corresponding to the parameters, described in the caption of Fig. 4.

Of course, the prediction depends on the accuracy of other parameters (refractive indices, $n_o$, $n_e$, thickness of the cell, *D*, rotational viscosity, $\gamma _1$) that should therefore be known as precisely as possible. For example, the cell thickness and the refractive indices explicitly determine the phase difference between ordinary and extraordinary polarization and thereby the magnitude of the light intensity, whereas the rotational viscosity directly scales with the elastic constants and in turn with the *I*(*t*) curves in time. In Fig. 4, we show the distributions of the elastic constants determined by the neural network that was trained on the data set corresponding to $D= 15.0 \ \upmu \text {m}$, $n_o=1.523$, $n_e=1.744$, $\gamma _1=0.20\ \text {Pa s}$ and tested for time-dependent transmittances that were simulated in systems with elastic constants $K_{11}=11.0 \text { pN}$, $K_{33}=17.0 \text { pN}$, but slightly modified parameters *D*, $n_o$, $n_e$, $\gamma _1$. Training a neural network with data corresponding to the inaccurate thickness of the cell *D* or refracitve indices $n_o$, $n_e$ causes the predicted elastic constants to be in the wrong ratio, while the inaccuracy of the rotational viscosity $\gamma _1$ causes that both predicted constants are shifted by the same factor.

Once the neural network is well-trained from numerically generated data pairs (${\textbf{X}},{\textbf{T}}$) calculated with the same thickness of the cell *D*, refractive indices $n_o$, $n_e$ and the rotational viscosity $\gamma _1$ as used in the experimental setup, the trained network can be utilized to predict elastic constants of a real sample from an experimentally measured *I*(*t*) as well.

**Figure 5:**
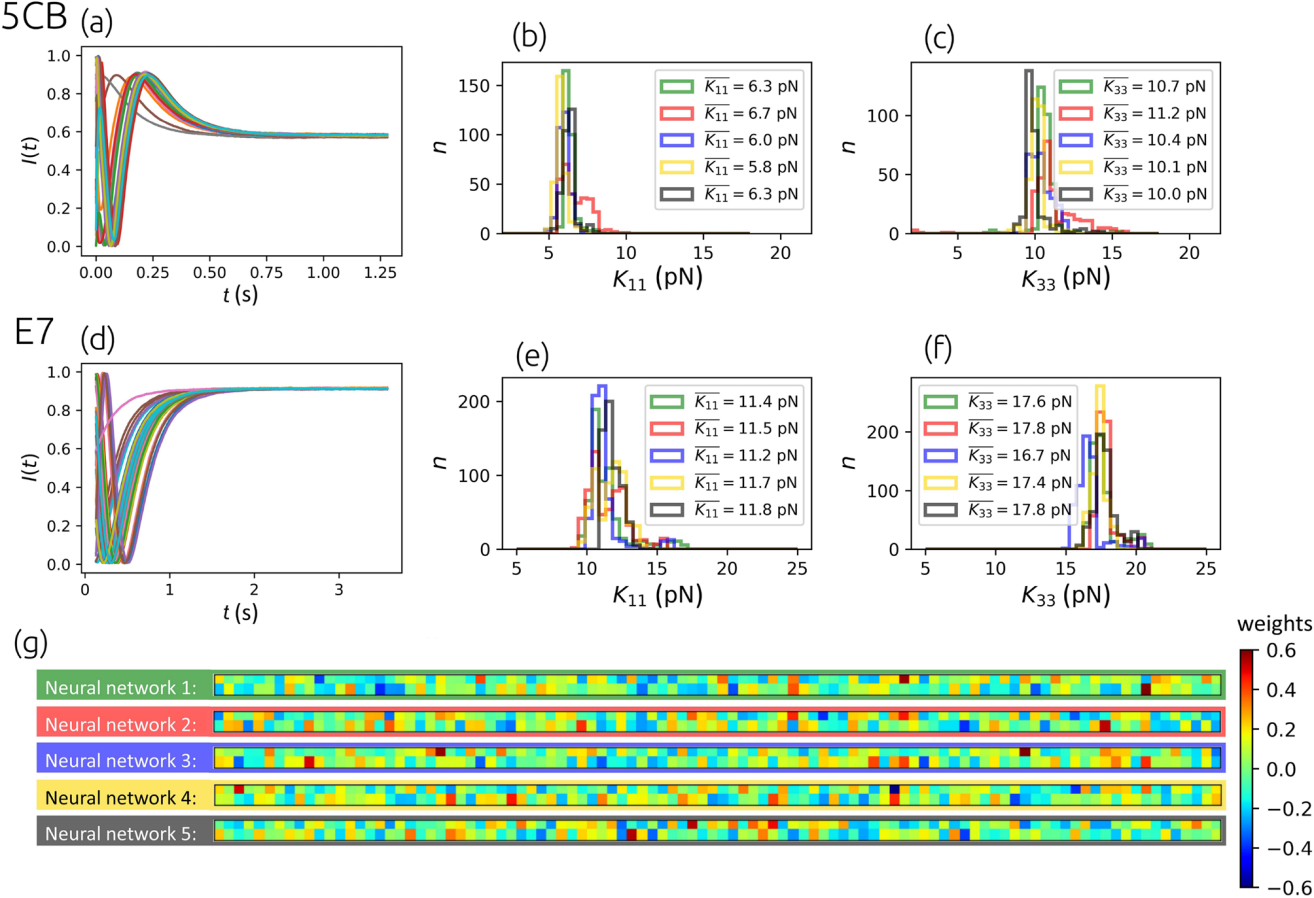
Results from experimentally measured data. Panels (**a**) and (**d**) show the measurements of the time-dependent intensities *I*(*t*) in 5CB and E7 at room temperature. Different curves correspond to different initial states of the director $\theta (z,t=0)$ caused by random electric pulses. Panels (**b**), (**c**), (**e**), (**f**) show the distributions of elastic constants of 5CB and E7 determined by five independently trained neural networks for each material. The following parameters were used to build the training data sets in accordance with the experimental setup: (**i**) 5CB setup has $D=10\ \upmu \text {m}$, $\gamma _1=0.098\ \text {Pa s}$^37,38^, $n_o=1.5450$, $n_e=1.7400$^46–48^, $\overline{\lambda }=505\text { nm}$, $\sigma _\lambda =20\ \text {nm}$, $K_{11}$ and $K_{33}$ range in the training data set $U(2\text { pN},\ 18\text { pN})$, and (ii) E7 setup has $D=15\ \upmu \text {m}$, $\gamma _1=0.200\ \text {Pa s}$^49^, $n_o=1.5225$, $n_e=1.7435$^48^, $\overline{\lambda }=595\text { nm}$, $\sigma _\lambda =8\ \text {nm}$, $K_{11}$ and $K_{33}$ range in the training data set $U(5\text { pN},\ 25\text { pN})$. Panel (**g**) shows the values of transposed matrices of weights between the last hidden layer and the output layer of five independently trained neural networks that were used to predict elastic constants of the E7 sample. All five neural networks had the same architecture and identical training hyperparameters (learning rate, batch size, number of epochs, etc.), and the same training data were used. This illustrates that there exist, due to a large number of model parameters (weights and biases) and random initialization of them, many neural networks with completely different combinations of weights that still result in very similar outputs of the network.

#### Method validation: prediction of $K_{11}$ and $K_{33}$ from experimental data

Experimentally, the nematic liquid crystal samples of 5CB and E7 were used in $D=10.0\ \upmu \text {m}$ and $D=15.0\ \upmu \text {m}$ thick cells, respectively, with strong uniform planar anchoring at the bottom ($z=0$) and homeotropic anchoring at the top surface ($z=D$) in both cases. The sample of the NLC between crossed polarizers was illuminated by a lamp with a known spectrum and the transmitted light was measured. First, using transparent electrodes positioned at the boundaries of the cell, the director was randomly deformed within the *x*-*z* plane by the pulses of the electric field at times $t<0$ and after the voltage was turned off at $t=0$, the director freely relaxed from its randomly deformed initial state to the minimum free energy state. 30 experimental measurements *I*(*t*) were done for each material and then normalized to the interval [0, 1] and interpolated to 500 time steps within the same time domain as numerically simulated intensities that were used for training, $[0,T=499\Delta t]$. Since the performance of our method does not depend on the initial state of the director – due to the variety of the director initial states used for the training data set – the transmittance *I*(*t*) could be measured after the sample was already partially relaxed. This allows us that the measured intensities *I*(*t*) can be interpolated to many random intervals $[\Lambda ,\Lambda +T]$, where $\text {max}(\Lambda )=T/10$, to increase the number of inputs $\textbf{X}$ and consequently get more results ${\textbf{Y}}=[K_{11}$, $K_{33}]$ to achieve better statistics of the predicted values.

**Figure 6:**
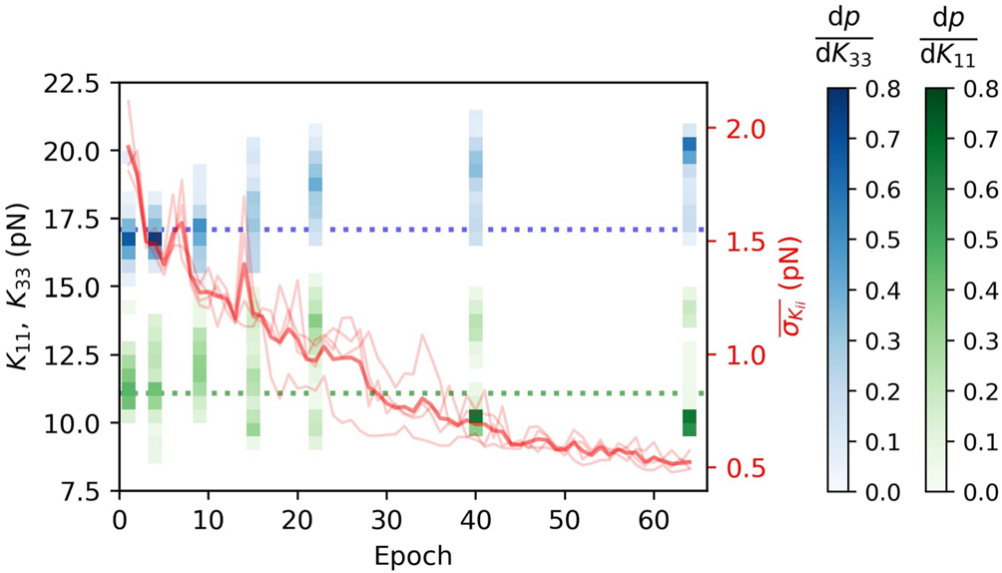
Possible overfitting in the extraction of elastic constants from experimental data. Note how the accuracy of the method improves with the number of epochs for numerical validation data (in red, right axis), but can lose accuracy for a high number of epochs on experimental data (for epochs $\gtrsim 10$, in green and blue histograms, left axis). Dotted horizontal lines show the anticipated values of the elastic constants from the literature for E7. $\overline{\sigma _{ K_{ii}}}$ is mean absolute error of predicted elastic constants corresponding to the numerical examples from the validation set; the solid line represents the average that generally decreases from epoch to epoch. The probability density functions of predictions $\text {d}p/\text {d} K_{11}$ and $\text {d}p/\text {d} K_{33}$ after 1, 4, 9, 15, 22, 40, 64 epochs are shown. Bin width is 0.5 pN.

The measured time-dependent intensities and distributions of predicted elastic constants for the 5CB and the E7 liquid crystal samples at room temperature (approx. 23 °C) are shown in Fig. 5. In each histogram, there are five different distributions of predictions for $K_{11}$ and $K_{33}$ made by five differently trained neural networks. The weights and biases of the networks are always initially set to random values^42^ and since the number of them is very large, their final values are different after each training, but the predictions are generally still similar. The transposed matrices of the values of weights (of size $100\times 2$) between the last hidden layer (of size 100) and the output layer (of size 2) from the networks predicting elastic constants of the E7 sample are shown in the panel (g) of Fig. 5. The elastic constants determined by neural networks are compared to the values published in the literature in Table 1. As illustrated in Fig. 4, the accuracy of the predicted elastic constants depends on the accuracy of different material and geometrical parameters, such as *D*, $n_o$, $n_e$, and $\gamma _1$. In the used (experimental) setup, the thickness of the cell was measured to $\pm 0.1\, \upmu$m accuracy, the refractive indices to $\pm 0.001$ accuracy, in agreement with typical values reported in the literature^46–48^. Rotational viscosity $\gamma _1$ was taken from the literature^37,38,49^.

**Table 1:** Comparison of our results from experimentally measured data to the values published in the literature.

		$K_{11}$	$K_{33}$
*5CB *			
Our results		6.2 pN ± 0.6 pN	10.5 pN ± 1.1 pN
Literature	^50^	6.5 pN	9.8 pN
	^36^	6.6 pN	9.0 pN
*E7 *			
Our results		11.5 pN ± 1.2 pN	17.4 pN ± 0.9 pN
Literature	^51^	11.1 pN	17.1 pN
	^52^	12 pN	17 pN

By increasing the number of training epochs, the mean absolute error of predicted constants for numerical examples from the validation set decreases, as shown by the red curve in Fig. 6, but the predictions from experimental measurements of the transmittance *I*(*t*) become increasingly inaccurate, compared to the expected values^51^, $K_{11}\approx 11.1\ \text {pN}$, $K_{33}\approx 17.1\ \text {pN}$, as shown by blue and green histograms in Fig. 6. Besides that, multiple peaks can emerge in the distributions of elastic constants determined by neural networks. We speculate that the reason is the following. Differences between the measured and the simulated time-dependent intensities are inevitable: There are always inaccuracies in the parameters (*D*, $n_o$, $n_e$, $\gamma _1$) that are used to generate the training data, the backflow and the light scattering are neglected in simulations, there is also some noise in the experimental measurements. Therefore, we can only approximately simulate the transmittances *I*(*t*). Besides that, it is known that neural networks can become overfitted^42,53^ and therefore less general and more sensitive to details and noise during training with the increasing number of training epochs. We observe that as long as the model is general enough (after less than $\sim 10$ epochs), the distributions of the predicted constants are broader but centered near the actual constants, while after many ($\gtrsim 20$) epochs, the distributions of the predictions get narrower but less accurate. For this reason, to achieve optimal predictions, one should not over-train the network when the aim is to predict material parameters from experimentally measured signals that are, in details, always slightly different from the simulated ones. Therefore, to avoid overfitting, we stopped training after 10 or even fewer epochs of training. In Fig. 5 and in Table 1, the predicted elastic constants were determined by models that were trained in 5 epochs.

**Figure 7:**
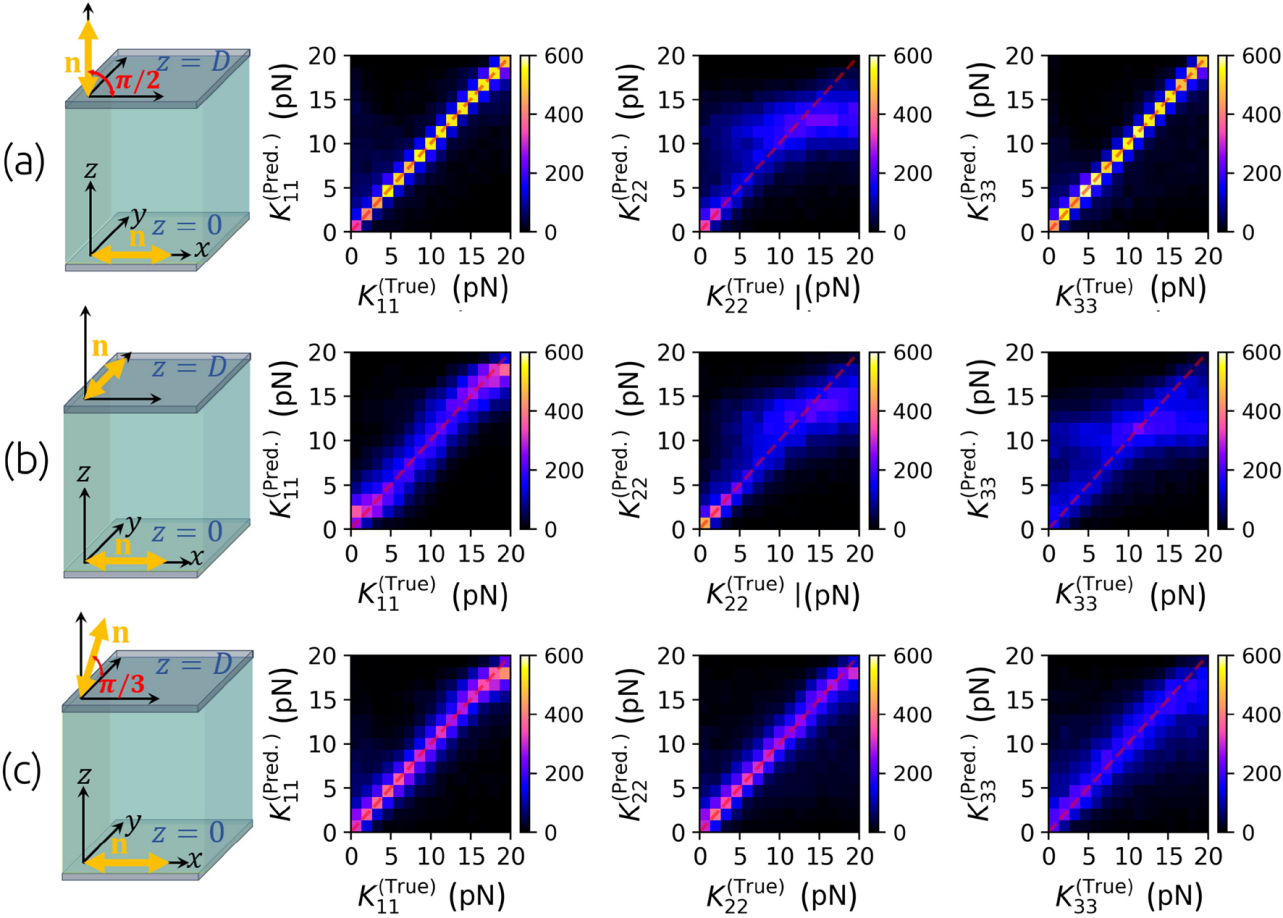
Prediction of all three elastic constants from *I*(*t*) measured through samples with 3D director with 1D dependence, $\textbf{n}(z,t)=(n_x(z,t),n_y(z,t),n_z(z,t))$. Comparison of predicted and actual elastic constants $K_{11}$, $K_{22}$, $K_{33}$ from the numerical validation data sets with different anchoring combinations, marked by two-headed arrows in the first column. While in the hybrid-aligned nematic (HAN) cell (**a**), it is possible to determine $K_{11}$ and $K_{33}$, in the twisted nematic (TN) cell (**b**), only $K_{11}$ can be roughly predicted, but using a tilted twist nematic (TTN) cell with planar ($\theta (z=0)=0$, $\phi (z=0)=0$) and tilted ($\theta (z=D)=\pi /3$, $\phi (z=D)=\pi /2$) anchoring (**c**), it is achievable to extract all three constants from the time-dependent transmittance *I*(*t*) at once.

#### Determining $K_{11}$, $K_{22}$ and $K_{33}$

To extract all three elastic constants $K_{11}$, $K_{22}$ and $K_{33}$ from *I*(*t*), it proves that more complex deformations of the director that include twist are needed to emerge in the measuring cell or geometry. To keep the cell geometry, therefore, the director parametrization $\textbf{n}(z,t)=(n_x(z,t),n_y(z,t),n_z(z,t))=(\cos \phi (z,t)\cos \theta (z,t),\sin \phi (z,t)\cos \theta (z,t),\sin \theta (z,t))$ with infinitely strong anchoring that gives fixed boundary conditions $\phi (z=0,t)$, $\theta (z=0,t)$, $\phi (z=D,t)$, $\theta (z=D,t)$ is assumed. The initial state of the director in numerical simulations is then determined by two random functions $\theta (z,t=0)$ and $\phi (z,t=0)$. The transmittance is again simulated similarly using the Jones formalism, and the training of neural networks is almost the same as for the determination of two constants except for the output vector that is, in this case, 3-dimensional (for the three elastic constants), ${\textbf{T}}_{i}=[K_{11}^i,K_{22}^i,K_{33}^i]$.

As it is shown in Fig. 7, we have found out that in hybrid aligned nematic (HAN) cells (panel (a)) and in twisted nematic (TN) cells (panel (b)), determination of $K_{11}$, $K_{22}$ and $K_{33}$ at once is not possible, while in a tilted twist nematic (TTN) cell with planar ($\theta (z=0)=0$, $\phi (z=0)=0$) and tilted ($\theta (z=D)=\pi /3$, $\phi (z=D)=\pi /2$) anchoring (panel (c)), the *I*(*t*) apparently carries information about all three constants, that can therefore be determined by a properly trained neural network. The experimental realization of such cells is beyond the scope of this work, but is clearly realizable^54^.

### Determination of initial director configurations

As an alternative use of our method, if all material parameters — including elastic constants — are known, our neural networks methodology can also be used to predict the initial director field $\textbf{n}(z,t=0)$, from the time-dependent transmittance *I*(*t*). Below, we show results for effective 2D director geometry with $\textbf{n}(z,t)=(n_x(z,t),0,n_z(z,t))=(\cos \theta (z,t),0,\sin \theta (z,t))$.

Using a data set of 390,000 pairs $(\textbf{X}, \textbf{T})=\left( [I(t)],[\theta (z,t=0)]\right) =([I(t=0),I(t=\Delta t), ..., I(t=499 \Delta t)], [\theta (z=0,t=0),\theta (z=h,t=0),...,\theta (z=199h,t=0)])$, that is created in a similar way as in “Neural networks based method for determining elastic constants” section but with fixed elastic constants, we train the neural network with an input layer of size 500 and four fully-connected hidden layers built of 800, 600, 400, 200 neurons with rectified linear unit (ReLU) activation functions and an output layer of size 200 with linear activation function. To train the network, the mean absolute error of the predicted $\theta (z,t=0)$ is minimized.

**Figure 8:**
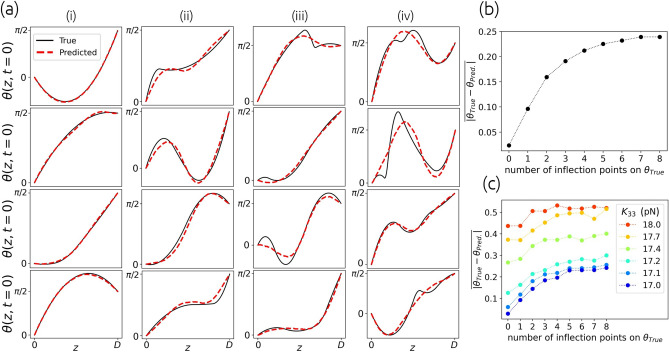
Determination of initial non-equilibrium state of the director $\textbf{n}(z,t=0)=(\cos \theta (z,t=0),0,\sin \theta (z,t=0))$ from the time-dependent transmittance *I*(*t*) for a NLC with elastic constants $K_{11}=11\ \text {pN}$, $K_{33}=17\ \text {pN}$. Column (i) in panel (**a**) shows examples with 0 inflection points on actual $\theta _\text {True}(z,t=0)$ profiles, in column (ii) there is 1 inflection point on each $\theta _\text {True}(z,t=0)$, etc. Panel (**b**) shows the mean absolute error of predicted $\theta _\text {Pred.}(z,t=0)$ that is lowest for “simple” curves without inflection points and increases with their number. The mean error is defined as $|\theta _\text {True}-\theta _\text {Pred.}|_i =1/N_i\sum _{n=1}^{N_i}\left( 1/N_r\sum _{j=1}^{N_r}\left| \theta _j^\text {True}-\theta _j^\text {Pred.}\right| \right)$, where $N_i$ is the number of validation pairs with *i* inflection points, and $N_r=200$ is the number of discretization points. If the trained neural network is used for a nematic sample with slightly different elastic constants, the recognition of profiles $\theta _\text {Pred.}(z,t=0)$ becomes less accurate, as shown in panel (**c**).

In Fig. 8, we compare neural network predictions of initial states of the director, described by $\theta (z,t=0)$, with actual ones for differently complex initial states, where the complexity is quantified by the number of inflection points in profiles. It is shown in panel (b) that this method works well when the actual $\theta _\text {True}(z,t=0)$ has zero inflection points, while the increasing number of inflection points results in an increasing prediction mean absolute error, but as shown in examples in panel (a) in Fig. 8, the approximate shape can still be determined. Like the method for determining the elastic constants, this one is also sensitive to the inaccuracy of the input parameters using which we generate the training set. This is illustrated in panel (c), which shows mean absolute errors of predictions for examples that were generated with slightly different elastic constant $K_{33}$ comparing to $K_{33}=17$ pN used for the generation of training data. Similar discrepancy is expected when other parameters (cell thickness, rotational viscosity, refractive indices) used for the creation of training data are different from the actual ones. However, knowing all material parameters, one could use neural networks to determine the initial structure of a nematic liquid crystal from the time-dependent transmittance *I*(*t*) measured during the reconfiguration of the NLC to the equilibrium. In experiments, this could allow for the fast automatic determination of the director field that is a result of imposed external fields, including electric, magnetic, or light.

## Conclusions

In conclusion, we have presented a method based on machine learning that can be used to determine selected material parameters, specifically the nematic elastic constants. Possible limitations of the method were analyzed, especially, the sensitivity on the values of other – to be known – material parameters. The presented approach of combining numerical simulations and experimental measurements by supervised machine learning and neural networks could also be generalized to determine other parameters of liquid crystals in nematic or other phases, such as Leslie viscosities, birefringence, dielectric anisotropy, anchoring strength, probably using more complex nematic director geometries. There are also no principal limitations for using a conceptually similar approach for determining selected dynamic or static parameters – such as tumbling parameter or degree of order coupling terms – for which established experimental methods are very scarce or do not even exist, including in passive, active, or biological soft matter.

## Methods

### Nematic and light transmission modeling

The established approach to characterization of nematic orientational order at the mesoscopic scales is by the construction of the total free energy functional^55^. At temperatures below the temperature of the nematic–isotropic phase transition and in the absence of external forces due to external fields or surface anchoring, the leading mechanism that affects the nematic ordering is nematic elasticity with any deformation of the orientational order from the unifrom state increasing the elastic free energy of the system. In the Frank-Oseen formulation, that is based on the director field $\textbf{n}$, which has $\textbf{n} \rightarrow -\textbf{n}$ symmetry, the elastic free energy can be written as $F_\text {FO}=\int f_\text {FO}\text {d} V$, where $f_\text {FO}$ is the Frank-Oseen elastic free energy density

1 $$\begin{aligned} f_\text {FO}=\frac{K_{11}}{2}(\nabla \cdot \textbf{n})^2 +\frac{K_{22}}{2}(\textbf{n}\cdot \nabla \times \textbf{n})^2 +\frac{K_{33}}{2}(\textbf{n}\times \nabla \times \textbf{n})^2, \end{aligned}$$

and $K_{11}$, $K_{22}$ and $K_{33}$ are the nematic elastic constants. The $K_{11}$, $K_{22}$, and $K_{33}$ terms describe the increase of the free energy due to the splay, twist and bend deformations, respectively. The equilibrium configuration of the director $\textbf{n}(\textbf{r})$ with minimum full elastic free energy $F_{FO}$ in the absence of external electric or magnetic fields can be found by solving Euler-Lagrange (EL) equations $h_i=\frac{\partial }{\partial x_j}\frac{f_{FO}}{\partial \left( \frac{\partial n_i}{\partial x_j}\right) }-\frac{\partial f_{FO}}{\partial n_i}=0,$ where $\textbf{h}=(h_x,h_y,h_z)$ is the molecular field which vanishes in the equilibrium. In principle, free energy (Eq. 1) could also include saddle-splay $f_{24}=-K_{24}(\nabla \cdot (\textbf{n} (\nabla \cdot \textbf{n})+\textbf{n} \times (\nabla \times \textbf{n})))$ and splay-bend $f_{13}=K_{13}(\nabla \cdot (\textbf{n} (\nabla \cdot \textbf{n})))$ free energy contributions. However, these free energy contributions are relevant only through the boundary conditions^35,56^, and thus in simple geometries can usually be ignorred^57^. Furthermore, especially in nematic geometries, which include topological defects, the tensorial Landau-de Gennes formulation of the free energy is more appropriate to use^40,58^, and actually, the developed method could also be extended to such tensorial modeling of the nematic. We model the relaxation of the nematic from an arbitrary initial configuration to the equilibrium by the simplified relaxational dynamics equations:

2 $$\begin{aligned} \gamma _1\frac{\partial n_i}{\partial t}=h_i, \qquad i=x,y,z, \end{aligned}$$

where $\gamma _1$ is the rotational viscosity, which notably ignores material flow and the corresponding backflow coupling^59–62^. Such simplified dynamics is an approximation, but when compared to experiments, it often proves sufficient in confined systems and simpler geometries, where weak material flow or flow with a simple spatial profile can develop.

Due to their anisotropic structure, liquid crystals are optically birefringent, and consequently light which is traveling through them can change its phase, polarization, and direction of propagation. The latter can be neglected if the liquid crystal sample is thin and if the transmitted light intensity is measured close to the sample. In this case, the change in polarization of light when passing through a liquid crystal can be described by the basic Jones matrix formalism, where the liquid crystal acts as a phase retarder. If it is placed between crossed polarizers, its director field configuration can affect the phase change and thus the intensity of the transmitted light.

### Experimental measurement

In the experiments, we have used cells with hybrid alignment nematic configuration where the bottom glass was covered with rubbed polyimide (SE-5291, Nissan) to achieve planar alignment and the second glass was covered with DMOAP silane (ABCR GmbH), which ensures perpendicular orientation at the top glass. The thickness of the cells was controlled with Mylar spacers and measured by the standard interferometric method using a spectrophotometer. ITO coated glasses were used that external electric field perpendicular to substrates was applied. The cells were filled with nematic liquid crystals 5CB or E7 using a capillary effect. In the experiment, we have measured the intensity of transmitted light through the LC sample placed between the crossed polarizers using the optical microscope with 20x objective. The easy axis of the planar anchoring at the bottom surface was set at the angle 45° relative to polarizers. The sample was illuminated with two different LEDs with 505 and 590 nm (Thorlabs M505L3 and M590L4). Different starting position of the director profile was achieved by applying 100 ms electric pulse with a random shape using a programmable waveform generator (DG1022Z, Rigol). The transmitted intensity was measured with a photodiode (SM05PD1A, Thorlabs) and amplifier (PDA200C, Thorlabs) in combination with a digital oscilloscope (MS09404A, Agilent) and digital delay generator (DG645, Stanford research systems). The temperature of the samples was kept constant using a home-made heating stage.

## Data Availability

Sample data supporting this study’s findings which are used for training and testing neural networks in the cited Jupyter Notebook tutorial is archived in Zenodo^65^. Complete data sets are available upon reasonable request from the first author J.Z.

## Code availability

The method with relevant code is publicly available in an interactive Jupyter Notebook tutorial on Google Colab^63^ and archived in Zenodo^64^.
